# On the Wiener Polarity Index of Lattice Networks

**DOI:** 10.1371/journal.pone.0167075

**Published:** 2016-12-08

**Authors:** Lin Chen, Tao Li, Jinfeng Liu, Yongtang Shi, Hua Wang

**Affiliations:** 1 Center for Combinatorics and LPMC-TJKLC, Nankai University, Tianjin, China; 2 College of Computer and Control Engineering, Nankai University, Tianjin 300071, China; 3 Department of Mathematical Sciences Georgia Southern University, Statesboro, GA 30460-8093, United States of America; Tianjin University of Technology, CHINA

## Abstract

Network structures are everywhere, including but not limited to applications in biological, physical and social sciences, information technology, and optimization. Network robustness is of crucial importance in all such applications. Research on this topic relies on finding a suitable measure and use this measure to quantify network robustness. A number of distance-based graph invariants, also known as topological indices, have recently been incorporated as descriptors of complex networks. Among them the Wiener type indices are the most well known and commonly used such descriptors. As one of the fundamental variants of the original Wiener index, the Wiener polarity index has been introduced for a long time and known to be related to the cluster coefficient of networks. In this paper, we consider the value of the Wiener polarity index of lattice networks, a common network structure known for its simplicity and symmetric structure. We first present a simple general formula for computing the Wiener polarity index of any graph. Using this formula, together with the symmetric and recursive topology of lattice networks, we provide explicit formulas of the Wiener polarity index of the square lattices, the hexagonal lattices, the triangular lattices, and the 3^3^ ⋅ 4^2^ lattices. We also comment on potential future research topics.

## Introduction

Robustness is the ability of a network to maintain performance when encountering attacks or enduring partial failure. In order to decide whether a given network is robust, a way to quantitatively measure network robustness is needed. Once such a measure has been established, it can serve as a standard for comparing networks or a guidance for improving existing networks and designing new networks. Intuitively robustness is all about back-up possibilities, or alternative paths, but it is rather challenging to capture all these concepts in a simple mathematical formula. During the recent years a lot of robustness measures have been proposed by scientists from different backgrounds, including but not limited to Biology, Chemistry, Computer Science, Engineering, Physics, and Mathematics [1–7]. Generally a network is considered as a graph consisting of a set of vertices connected by edges, and the study of network robustness relies on the analysis of such underlying graphs.

One way to measure a network structure is through the so called structure descriptors, or topological indices [8]. In theoretical biology and chemistry, for instance, molecular structure descriptors are numerical parameters mathematically derived from the graph structure. They have been found to be useful for modeling physico-chemical, toxicologic, pharmacologic, biological and other properties of molecular compounds. These descriptors are mainly divided into three types: degree-based indices, distance-based indices and spectrum-based indices. Degree-based indices contain the (general) Randić index [9–11], the (general) zeroth order Randić index [12, 13], the Zagreb index [14, 15], the connective eccentricity index [16] and so on [17]. Distance-based indices [18] include the Balaban index [19, 20], the Wiener index and Wiener polarity index [21], the Kirchhoff index [22, 23] and so forth. Eigenvalues of graphs [24], various of graph energies [25–36], the HOMO-LUMO index [37, 38], and the Estrada index [39–41] belong to spectrum-based indices. There are also some topological indices defined based on both degrees and distances, such as the degree distance [42] and graph entropies [43, 44]. The study of mathematical properties of such graph indices and the evaluation of them in various graph structures have been of tremendous interest to researchers.

A *lattice graph*, or simply a *lattice*, is a graph possessing a drawing whose embedding in a Euclidean space $R^{n}$ forms a regular tiling. Because of the symmetric nature of its topology, lattice graphs appear to be among the most common network structures. For exactly the same reason, the computation of various physical and chemical indices of various lattice graphs has attracted the attention of many scientists as well as mathematicians. See, for instance, [45–51]. We will focus on the square lattices, the hexagonal lattices, the triangular lattices, and the 3^3^ ⋅ 4^2^ lattices, each corresponding to a grid with specific geometric shapes.

In this paper all graphs under consideration are finite, connected, undirected and simple. For standard notations and terminologies we follow [52]. Let *G* be a graph with vertex set *V*(*G*) and edge set *E*(*G*). The *distance* *d*_*G*_(*u*, *v*) (or simply *d*(*u*, *v*) when there is no confusion) between two vertices *u* and *v* of *G* is the length of the shortest path that connects *u* and *v*. One of the most well-known and well-studied distance-based graph indices is the *Wiener number* *W*(*G*), also termed as *Wiener index* in chemical or mathematical chemistry literatures. It is defined as the sum of distances over all unordered vertex pairs in *G* [21]. I.e.,

$$\begin{array}{c} W\left(G\right)=\sum_{\left\{u,v\}\subseteq V(G\right)}d_{G}\left(u,v\right). \end{array}$$

As a representative of successful structure-descriptors, the Wiener index has received much attention. For further details we refer the readers to some recent papers [53–56] and the comprehensive survey of Dobrynin, Entringer and Gutman [57].

Another important molecular descriptor was also introduced by Wiener [21], called the *Wiener polarity index*. Denoted by *W*_*p*_(*G*), it is defined as the number of unordered pairs of vertices that are at distance 3 in *G*. That is,

$$\begin{array}{c} W_{p}\left(G\right)=\left|\left\{\left(u,v\right)|d_{G}\left(u,v\right)=3,u,v\in V\left(G\right)\right\}\right|. \end{array}$$ (1)

In organic compounds, say paraffin, the Wiener polarity index is the number of pairs of carbon atoms which are separated by three carbon-carbon bonds. Based on the Wiener index and the Wiener polarity index, the formula

$$\begin{array}{c} t_{B}=aW\left(G\right)+bW_{p}\left(G\right)+c, \end{array}$$

was used to calculate the boiling points *t*_*B*_ of the paraffins, where *a*, *b* and *c* are constants for a given isomeric group.

In an acyclic structure, the Wiener polarity index can be expressed in terms of vertex degrees (see, for instance, Lemma 2). This unique characteristic of the Wiener polarity index makes it interesting for studies from both distance-based and degree-based points of view. However, compared with the Wiener index, surprisingly little attention has been paid to the Wiener polarity index until very recently. Nevertheless, the study of the Wiener polarity index has indeed caught the attention of many researchers. By using the Wiener polarity index, Lukovits and Linert demonstrated quantitative structure-property relationships in a series of acyclic and cycle-containing hydrocarbons in [58]. Hosoya in [59] found a physical-chemical interpretation of *W*_*p*_(*G*). Du et al. [60] described a linear time algorithm for computing the Wiener polarity index of trees and characterized the trees maximizing the index among all the trees of the given order. Later, Deng, Xiao and Tang characterized the extremal trees with respect to this index among all trees of order *n* and diameter *k* [61]. While for cycle-containing graphs, the maximum Wiener polarity index of unicyclic graphs and the corresponding extremal graphs were determined in [62]. In [63] Ma et al. determined the sharp upper bound of the Wiener polarity index among all bicyclic networks based on some graph transformations. Moreover, the extremal values of catacondensed hexagonal systems, hexagonal cacti and polyphenylene chains with respect to the Wiener polarity index were computed in [64]. It was proved that the Wiener polarity index of fullerenes with *n* carbon atoms is (9*n* − 60)/2 in the same paper. Recently, Hua and Das [65] established an upper bound on the Wiener polarity index in terms of Hosoya index and characterized the corresponding extremal graphs. They also obtaind Nordhaus-Gaddum-type results for *W*_*p*_(*G*). Other recent work on Wiener polarity index can be found in [66–70].

In this paper we study the Wiener polarity index of several common classes of lattices. In the section of Preliminaries and the square lattices, we first introduce some notations and previously established fundamental results on the Wiener polarity index of graphs. Through the concept of 3rd neighborhoods, we provide a simple but extremely useful formula for calculating the Wiener polarity index. We also present the computation of the Wiener polarity index of square lattices as an example of applications of such results. In the sections that follow, we discuss in details the computation of the Wiener polarity index of the hexagonal lattices, the triangular lattices, and the 3^3^ ⋅ 4^2^ lattices. We summarize our findings and propose some future directions of research in the section of Concluding remarks.

## Preliminaries and the square lattices

In this section, we first recall some notations. Then a general formula is presented for the Wiener polarity index of any graph. In mathematics, a *Cartesian product* is a mathematical operation which returns a product set (or simply product) from multiple sets. Given sets *A* and *B*, the Cartesian product of *A* and *B*, generally denoted by *A*□*B*, is the set of all ordered pairs (*a*, *b*), where *a* ∈ *A* and *b* ∈ *B*. That is,

$$\begin{array}{c} A□B=\{(a,b)|a\in A,b\in B\}. \end{array}$$

Given graphs *G* and *H* with vertex sets *U* and *V*, the *Cartesian product* *G*□*H* of graphs *G* and *H* is a graph such that the vertex set of *G*□*H* is *U*□*V*, and any two vertices (*u*, *u*′) and (*v*, *v*′) are adjacent in *G*□*H* if and only if either *u* = *v* and *u*′ is adjacent to *v*′ in *H*, or *u*′ = *v*′ and *u* is adjacent to *v* in *G*.

For a graph *G* and vertex *v* ∈ *V*(*G*), let *N*_*G*_(*v*) denote the neighborhood of *v* and *d*_*G*_(*v*) = |*N*_*G*_(*v*)| denote the degree of *v*. The greatest distance between any two vertices in *G* is the diameter of *G*, denoted by *diam*(*G*). The girth *g*(*G*) of *G*, is the length of a shortest cycle in *G*. For any integer *i*, we call $N_{G}^{i}\left(v\right)=\left\{u\in V\left(G\right)|d_{G}\left(u,v\right)=i\right\}$ the *ith neighborhood* of *v*, and the vertices in $N_{G}^{i}\left(v\right)$ are called the *ith neighbors* of *v*. In particular, $N_{G}^{1}\left(v\right)$ is precisely the neighborhood *N*_*G*_(*v*) of *v*, $N_{G}^{0}\left(v\right)=\left\{v\right\}$, while $N_{G}^{i}\left(v\right)=\emptyset$ for *i* > *diam*(*G*). In addition, let *P*_*n*_ and *C*_*n*_ denote the path and the cycle with *n* vertices, respectively.

The following lemmas are useful in the study of the Wiener polarity index of the lattice networks.

**Lemma 1** ([69]) *Let* *G* *and* *H* *be two non-trivial connected graphs, then*

$$\begin{array}{c} W_{p}\left(G□H\right)=W_{p}\left(G\right)|V\left(H\right)|+W_{p}\left(H\right)\left|V\left(G\right)\right|+2W_{2}\left(G\right)m\left(H\right)+2W_{2}\left(H\right)m\left(G\right), \end{array}$$

*where* *m*(*G*) *and* *m*(*H*) *are the number of edges of* *G* *and* *H*, *respectively, and* *W*_2_(*G*) = |{{*u*, *v*}|*d*(*u*, *v*) = 2, *u*, *v* ∈ *V*(*G*)}| *is the number of unordered pairs of vertices* {*u*, *v*} *of* *G* *such that* *d*_*G*_(*u*, *v*) = 2.

**Lemma 2** ([60]) *Let* *T* = (*V*, *E*) *be a tree. Then*

$$\begin{array}{c} W_{p}\left(T\right)=\sum_{uv\in E}\left(d_{T}\left(u\right)-1\right)\left(d_{T}\left(v\right)-1\right). \end{array}$$

**Lemma 3** ([62, 67]) *Let* *U* = (*V*, *E*) *be a unicyclic graph. Let* *C* *denote the unique cycle of* *U*:

- *If* *g*(*U*) = 3 *with* *V*(*C*) = {*v*_1_, *v*_2_, *v*_3_}, *then*
  $$\begin{array}{c} W_{p}\left(U\right)=\sum_{uv\in E}\left(d_{U}\left(u\right)-1\right)\left(d_{U}\left(v\right)-1\right)+9-2d_{U}\left(v_{1}\right)-2d_{U}\left(v_{2}\right)-2d_{U}\left(v_{3}\right); \end{array}$$
- *If* *g*(*U*) = 4 *with* *V*(*C*) = {*v*_1_, *v*_2_, *v*_3_, *v*_4_}, *then*
  $$\begin{array}{c} W_{p}\left(U\right)=\sum_{uv\in E}\left(d_{U}\left(u\right)-1\right)\left(d_{U}\left(v\right)-1\right)+4-d_{U}\left(v_{1}\right)-d_{U}\left(v_{2}\right)-d_{U}\left(v_{3}\right)-d_{U}\left(v_{4}\right); \end{array}$$
- *If* *g*(*U*)≥5, *then*
  $$\begin{array}{c} W_{p}\left(U\right)=\{\begin{array}{cc} \sum_{uv\in E}\left(d_{U}\left(u\right)-1\right)\left(d_{U}\left(v\right)-1\right)-5, & if\;g(U)=5,\\ \sum_{uv\in E}\left(d_{U}\left(u\right)-1\right)\left(d_{U}\left(v\right)-1\right)-3, & if\;g(U)=6,\\ \sum_{uv\in E}\left(d_{U}\left(u\right)-1\right)\left(d_{U}\left(v\right)-1\right),\;\;\;\; & if\;g(U)\geq 7. \end{array} \end{array}$$

Lastly, we provide a simple general formula for the Wiener polarity index of a graph, which plays an important role in the proofs of our main results.

**Lemma 4** *For any graph* *G*, *the Wiener polarity index* *W*_*p*_(*G*) *of* *G* *can be expressed as*

$$\begin{array}{c} W_{p}\left(G\right)=\frac{\sum_{v\in V(G)}\left|N_{G}^{3}\left(v\right)\right|}{2}. \end{array}$$ (2)

*Proof.* By the definitions of the Wiener polarity index and the *i*th neighborhood, we have

$$\begin{array}{c} W_{p}\left(G\right)=\left|\left\{\left(u,v\right)|d_{G}\left(u,v\right)=3,u,v\in V\left(G\right)\right\}\right| \end{array}$$

and

$$\begin{array}{c} |N_{G}^{3}\left(v\right)|=|\left\{\left(u,v\right)|d_{G}\left(u,v\right)=3,u\in V\left(G\right)\right\}|. \end{array}$$

Formula (2) then follows immediately.

To study the Wiener polarity index of lattice networks, we first consider the simple case concerning the square lattices.

Let *P*_*m*_□*P*_*n*_(*m* ≥ 2, *n* ≥ 2), *P*_*m*_□*C*_*n*_(*m* ≥ 2, *n* ≥ 3), and *C*_*m*_□*C*_*n*_(*m* ≥ 3, *n* ≥ 3) denote the square lattices with free, cylindrical and toroidal boundary conditions respectively, it is easy to see that *P*_*m*_□*P*_*n*_ is a sequence of spanning subgraphs of the sequence *P*_*m*_□*C*_*n*_ of finite graphs, and *P*_*m*_□*C*_*n*_ is a sequence of spanning subgraphs of the sequence *C*_*m*_□*C*_*n*_ of finite graphs. Following the aforementioned lemmas, the Wiener polarity index of the square lattices can be easily calculated. In the rest of this section, we assume without loss of generality, that *n* ≥ *m* in *P*_*m*_□*P*_*n*_ and *C*_*m*_□*C*_*n*_.

**Theorem 1** *Let* *P*_*m*_□*P*_*n*_, *P*_*m*_□*C*_*n*_ *and* *C*_*m*_□*C*_*n*_ *denote the square lattices with free, cylindrical and toroidal boundary conditions, respectively. Then*

$$\begin{array}{c} W_{p}\left(P_{m}□P_{n}\right)=\{\begin{array}{cc} 4n-10, & if\;m=2,n\geq 3;\\ 6mn-9m-9n+8, & if\;m\geq 3,n\geq 3; \end{array} \end{array}$$ (3)

$$\begin{array}{c} W_{p}\left(P_{m}□C_{n}\right)=\{\begin{array}{cc} 4n, & if\;m=2,n\geq 7;\\ 9m-21, & if\;m\geq 3,n=3;\\ 16m-32, & if\;m\geq 3,n=4;\\ 25m-45, & if\;m\geq 3,n=5;\\ 33m-54, & if\;m\geq 3,n=6;\\ 6mn-9n, & if\;m\geq 3,n\geq 7; \end{array} \end{array}$$ (4)

$$\begin{array}{c} W_{p}\left(C_{m}□C_{n}\right)=\{\begin{array}{cc} 9n, & if\;m=3,n\geq 7;\\ 16n, & if\;m=4,n\geq 7;\\ 25n, & if\;m=5,n\geq 7;\\ 33n, & if\;m=6,n\geq 7;\\ 6mn, & if\;m\geq 7,n\geq 7. \end{array} \end{array}$$ (5)

*Proof.* It follows from Lemma 2 that *W*_*p*_(*P*_2_) = 0 and *W*_*p*_(*P*_*m*_) = *m* − 3 for *m* ≥ 3. On the other hand, Lemma 3 yields that *W*_*p*_(*C*_3_) = *W*_*p*_(*C*_4_) = *W*_*p*_(*C*_5_) = 0, *W*_*p*_(*C*_6_) = 3, and *W*_*p*_(*C*_*n*_) = *n* for *n* ≥ 7. By the definition of *W*_2_(*G*), it is not difficult to see that *W*_2_(*P*_*m*_) = *m* − 2 for *m* ≥ 2, *W*_2_(*C*_3_) = 0, *W*_2_(*C*_4_) = 2, and *W*_2_(*C*_*n*_) = *n* for *n* ≥ 5. In addition, by Lemma 1 we have

$$\begin{array}{ccc} W_{p}\left(P_{m}□P_{n}\right) & = & W_{p}\left(P_{m}\right)|V\left(P_{n}\right)|+W_{p}\left(P_{n}\right)\left|V\left(P_{m}\right)\right|+2W_{2}\left(P_{m}\right)m\left(P_{n}\right)\\ & & +2W_{2}\left(P_{n}\right)m\left(P_{m}\right), \end{array}$$

proving Eq (3).

Similarly, Eqs (4) and (5) are also direct consequences of Lemma 1 and the above discussion.

**Remark 1** *For small values of* *m* *and* *n*, *still by Lemma* 1, *we have the followings*:

*W*_*p*_(*P*_2_□*P*_2_) = 0; *W*_*p*_(*P*_2_□*C*_3_) = 0, *W*_*p*_(*P*_2_□*C*_4_) = 4, *W*_*p*_(*P*_2_□*C*_5_) = 10, *W*_*p*_(*P*_2_□*C*_6_) = 18; *W*_*p*_(*C*_3_□*C*_3_) = 0, *W*_*p*_(*C*_3_□*C*_4_) = 12, *W*_*p*_(*C*_3_□*C*_5_) = 30, *W*_*p*_(*C*_3_□*C*_6_) = 45, *W*_*p*_(*C*_4_□*C*_4_) = 32, *W*_*p*_(*C*_4_□*C*_5_) = 60, *W*_*p*_(*C*_4_□*C*_6_) = 84, *W*_*p*_(*C*_5_□*C*_5_) = 100, *W*_*p*_(*C*_5_□*C*_6_) = 135, *W*_*p*_(*C*_6_□*C*_6_) = 180.

## The Wiener polarity index of the hexagonal lattices

**Fig 1:**
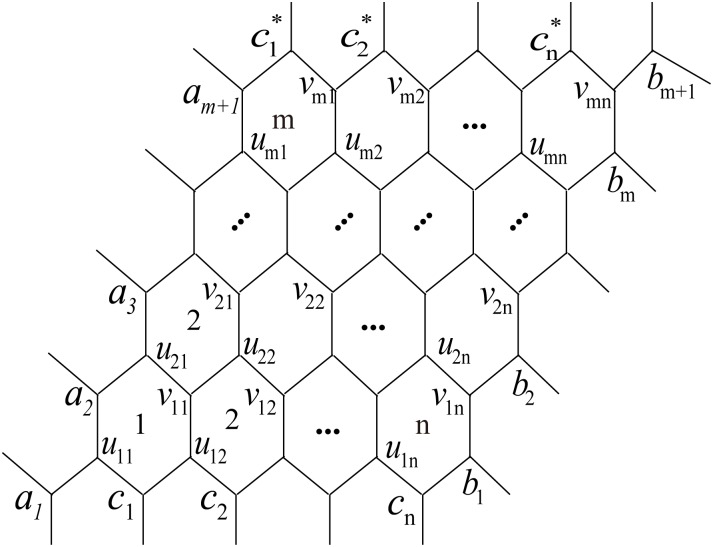
The hexagonal lattice.

Next we consider the Wiener polarity index of the hexagonal lattices. We follow the notations in [50]. The hexagonal lattices with toroidal, cylindrical and free boundary conditions, are denoted by *H*^*t*^(*n*, *m*), *H*^*c*^(*n*, *m*) and *H*^*f*^(*n*, *m*), respectively, where (*a*_1_, *b*_1_), (*a*_2_, *b*_2_), …, (*a*_*m*+1_, *b*_*m*+1_); $\left(a_{1},c_{1}^{*}\right)$, $\left(c_{1},c_{2}^{*}\right)$, $\left(c_{2},c_{3}^{*}\right)$, …, $\left(c_{n-1},c_{n}^{*}\right)$, (*c*_*n*_, *b*_*m*+1_) are edges in *H*^*t*^(*n*, *m*) (as illustrated in Fig 1). The hexagonal lattice *H*^*c*^(*n*, *m*) is obtained from *H*^*t*^(*n*, *m*) by deleting edges $\left(a_{1},c_{1}^{*}\right)$, $\left(c_{1},c_{2}^{*}\right)$, $\left(c_{2},c_{3}^{*}\right)$, …, $\left(c_{n-1},c_{n}^{*}\right)$, (*c*_*n*_, *b*_*m*+1_). If the edges (*a*_1_, *b*_1_), (*a*_2_, *b*_2_), …, (*a*_*m*+1_, *b*_*m*+1_) are also removed, then the hexagonal lattice *H*^*f*^(*n*, *m*) with free boundary condition is obtained. It is easy to see that |*V*(*H*^*t*^(*n*, *m*))| = |*V*(*H*^*c*^(*n*, *m*))| = |*V*(*H*^*f*^(*n*, *m*))| = 2(*n* + 1)(*m* + 1). Furthermore, from the definitions it is obvious that *H*^*f*^(*n*, *m*) and *H*^*c*^(*n*, *m*) are spanning subgraphs of *H*^*t*^(*n*, *m*). In the following result we assume, without loss of generality, that *m* ≥ *n* in *H*^*t*^(*n*, *m*) and *H*^*f*^(*n*, *m*).

**Theorem 2** *For the hexagonal lattices* *H*^*t*^(*n*, *m*), *H*^*c*^(*n*, *m*) *and* *H*^*f*^(*n*, *m*) *with toroidal, cylindrical and free boundary conditions, we have*

$$\begin{array}{c} W_{p}\left(H^{t}\left(n,m\right)\right)=\{\begin{array}{cc} 10(m+1), & if\;n=1,m\geq 3;\\ 24(m+1), & if\;n=2,m\geq 3;\\ 9(n+1)(m+1), & if\;n\geq 3,m\geq 3; \end{array} \end{array}$$

$$\begin{array}{c} W_{p}\left(H^{c}\left(n,m\right)\right)=\{\begin{array}{cc} 10m-4, & if\;n=1,m\geq 1;\\ 24m-3, & if\;n=2,m\geq 1;\\ 9m(n+1), & if\;n\geq 3,m\geq 1; \end{array} \end{array}$$

$$\begin{array}{c} W_{p}\left(H^{f}\left(n,m\right)\right)=9nm-2\;\;\;for\;n\geq 1,m\geq 1. \end{array}$$

**Fig 2:**
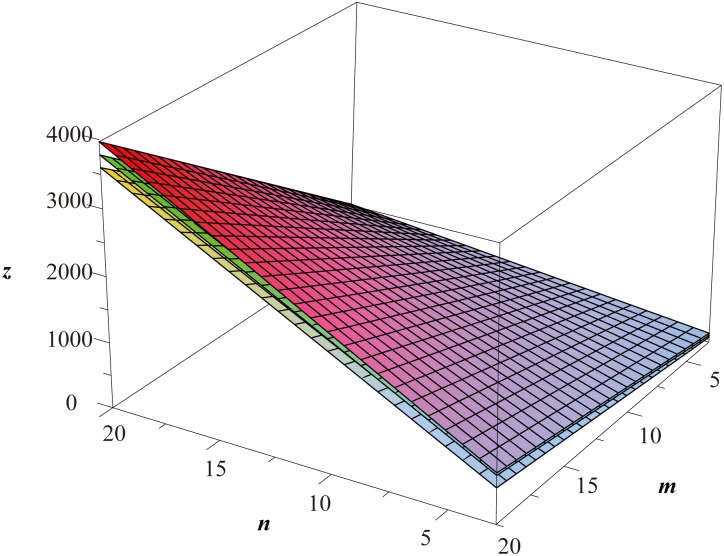
The Wiener polarity index of *H*^*t*^(*n*, *m*), *H*^*c*^(*n*, *m*) and *H*^*f*^(*n*, *m*).

**Remark 2** *As illustrated in* Fig 2, *we generally have*

$$\begin{array}{c} W_{p}\left(H^{t}\left(n,m\right)\right)>W_{p}\left(H^{c}\left(n,m\right)\right)>W_{p}\left(H^{f}\left(n,m\right)\right), \end{array}$$

*with the common asymptotic value* 9*mn* *as both* *m* *an* *n* *approaches infinity*.

*Proof.* We consider each of these three lattices through different cases.

**(1)** First consider *H*^*t*^(*n*, *m*). Our argument relies on the simple fact that every vertex in *H*^*t*^(*n*, *m*) has the same number of 3rd neighbors. Take, for instance, vertex *a*_1_ from Fig 1:

- If *n* = 1 and *m* ≥ 3, then
  $$\begin{array}{c} N_{H^{t}\left(1,m\right)}^{3}\left(a_{1}\right)=\left\{u_{21},b_{2},u_{m1},b_{m},b_{m+1}\right\}. \end{array}$$
  Hence the number of 3rd neighbors of any vertex in *H*^*t*^(1, *m*) is 5 and Lemma 4 implies that
  $$\begin{array}{c} W_{p}\left(H^{t}\left(1,m\right)\right)=\frac{\sum_{v\in H^{t}\left(1,m\right)}\left|N_{H^{t}\left(1,m\right)}^{3}\left(v\right)\right|}{2}=\frac{5\times 2(1+1)(m+1)}{2}=10\left(m+1\right). \end{array}$$
- If *n* = 2 and *m* ≥ 3, then
  $$\begin{array}{c} N_{H^{t}\left(2,m\right)}^{3}\left(a_{1}\right)=\left\{u_{21},b_{2},u_{12},c_{2}^{*},b_{m+1},u_{22},u_{m1},u_{m2}\right\}. \end{array}$$
  Thus the number of 3rd neighbors of any vertex in *H*^*t*^(2, *m*) is 8 and Lemma 4 implies that
  $$\begin{array}{c} W_{p}\left(H^{t}\left(2,m\right)\right)=\frac{\sum_{v\in H^{t}\left(2,m\right)}\left|N_{H^{t}\left(2,m\right)}^{3}\left(v\right)\right|}{2}=\frac{8\times 2(2+1)(m+1)}{2}=24\left(m+1\right). \end{array}$$
- If *n* ≥ 3 and *m* ≥ 3, then
  $$\begin{array}{c} N_{H^{t}\left(n,m\right)}^{3}\left(a_{1}\right)=\left\{u_{21},b_{2},u_{12},c_{2}^{*},u_{1n},b_{m+1},u_{2n},u_{m1},u_{m2}\right\}. \end{array}$$
  Similarly we have
  $$\begin{array}{ccc} W_{p}\left(H^{t}\left(n,m\right)\right) & = & \frac{\sum_{v\in H^{t}\left(n,m\right)}\left|N_{H^{t}\left(n,m\right)}^{3}\left(v\right)\right|}{2}=\frac{9\times 2(n+1)(m+1)}{2}\\ & = & 9(n+1)(m+1). \end{array}$$

**(2)** In the case of *H*^*c*^(*n*, *m*) (Fig 1), we can make similar observations on vertices whose 3rd neighborhood share the same cardinality. For this purpose we partition the vertex set *H*^*c*^(*n*, *m*) into *m* + 1 disjoint classes:

- $$\begin{array}{c} V_{0}=\left\{a_{1},c_{1},c_{2},\ldots ,c_{n},c_{1}^{*},c_{2}^{*},\ldots ,c_{n}^{*},b_{m+1}\right\}, \end{array}$$
- $$\begin{array}{c} V_{1}=\left\{u_{11},u_{12},\ldots ,u_{1n},b_{1},a_{m+1},v_{m1},v_{m2},\ldots ,v_{mn}\right\}, \end{array}$$
- $$\begin{array}{c} V_{2}=\left\{a_{2},v_{11},v_{12},\ldots ,v_{1n},u_{m1},u_{m2},\ldots ,u_{mn},b_{m}\right\},\\ \vdots \end{array}$$
- $$\begin{array}{c} V_{m}=\left\{u_{(\frac{m+1}{2})1},u_{(\frac{m+1}{2})2},\ldots ,u_{(\frac{m+1}{2})n},b_{\frac{m+1}{2}},a_{(\frac{m+1}{2})+1},v_{(\frac{m+1}{2})1},v_{(\frac{m+1}{2})2},\ldots ,v_{(\frac{m+1}{2})n}\right\} \end{array}$$
  when *m* is odd, or
  $$\begin{array}{c} V_{m}=\left\{a_{\frac{m}{2}+1},v_{\frac{m}{2}1},v_{\frac{m}{2}2},\ldots ,v_{\frac{m}{2}n},u_{(\frac{m}{2}+1)1},u_{(\frac{m}{2}+1)2},\ldots ,u_{(\frac{m}{2}+1)n},b_{\frac{m}{2}+1}\right\} \end{array}$$
  when *m* is even.

Since vertices in the same class have the same number of 3rd neighbors, we only need to consider one vertex from each class.

- If *n* = 1, it is easy to see that *W*_*p*_(*H*^*c*^(1, 1)) = 6 and *W*_*p*_(*H*^*c*^(1, 2)) = 16. When *m* ≥ 3, one can verify the followings:
  $$\begin{array}{c} N_{H^{c}\left(1,m\right)}^{3}\left(a_{1}\right)=\left\{u_{21},b_{2}\right\},\;\;\;\;\;\;N_{H^{c}\left(1,m\right)}^{3}\left(u_{11}\right)=\left\{a_{3},v_{11},v_{21}\right\}, \end{array}$$
  $$\begin{array}{c} N_{H^{c}\left(1,m\right)}^{3}\left(a_{2}\right)=\left\{u_{31},b_{3},b_{1}\right\},\;\;\;\;\;\;N_{H^{c}\left(1,m\right)}^{3}\left(u_{21}\right)=\left\{a_{1},c_{1},v_{21},a_{4},v_{31}\right\}, \end{array}$$
  $$\begin{array}{c} N_{H^{c}\left(1,m\right)}^{3}\left(a_{i}\right)=\left\{u_{(i-2)1},b_{i-1},b_{i-2},u_{(i+1)1},b_{i+1}\right\}\left(3\leq i\leq \lfloor \frac{m}{2}\rfloor +1\right), \end{array}$$
  $$\begin{array}{c} N_{H^{c}\left(1,m\right)}^{3}\left(u_{i1}\right)=\left\{a_{i-1},v_{(i-2)1},v_{i1},v_{(i+1)1},a_{i+2}\right\}\left(3\leq i\leq \lceil \frac{m}{2}\rceil \right). \end{array}$$
  That is, $|N_{H^{c}\left(1,m\right)}^{3}\left(v\right)|=2$ for *v* ∈ *V*_0_, $|N_{H^{c}\left(1,m\right)}^{3}\left(v\right)|=3$ for *v* ∈ *V*_1_ or *v* ∈ *V*_2_, and $|N_{H^{c}\left(1,m\right)}^{3}\left(v\right)|=5$ for any vertex *v* in *V*′ = *V*(*H*^*c*^(1, *m*)) − *V*_0_ − *V*_1_ − *V*_2_. By Lemma 4,
  $$\begin{array}{ccc} W_{p}\left(H^{c}\left(1,m\right)\right) & = & \frac{\sum_{v\in V(H^{c}\left(1,m\right))}\left|N_{H^{c}\left(1,m\right)}^{3}\left(v\right)\right|}{2}\\ & = & \frac{2\times |V_{0}\left|+3\times \right|V_{1}\left|+3\times \right|V_{2}\left|+5\times \right|V^{\prime }|}{2}\\ & = & \frac{2\times 4+3\times 4+3\times 4+5\times [4(m+1)-4\times 3]}{2}\\ & = & 10m-4. \end{array}$$
  It is not hard to check that *W*_*p*_(*H*^*c*^(1, 1)) (with *m* = 1) and *W*_*p*_(*H*^*c*^(1, 2)) (with *m* = 2) also satisfy this expression.
- If *n* = 2, then *W*_*p*_(*H*^*c*^(2, 1)) = 21 and *W*_*p*_(*H*^*c*^(2, 2)) = 45. When *m* ≥ 3, the 3rd neighborhoods of the representative vertices are
  $$\begin{array}{c} N_{H^{c}\left(2,m\right)}^{3}\left(a_{1}\right)=\left\{u_{21},b_{2},u_{12},u_{22}\right\}, \end{array}$$
  $$\begin{array}{c} N_{H^{c}\left(2,m\right)}^{3}\left(u_{11}\right)=\left\{a_{3},v_{11},v_{12},v_{22},c_{2}\right\}, \end{array}$$
  $$\begin{array}{c} N_{H^{c}\left(2,m\right)}^{3}\left(a_{2}\right)=\left\{u_{31},b_{3},u_{12},u_{22},b_{1},u_{32}\right\}, \end{array}$$
  $$\begin{array}{c} N_{H^{c}\left(2,m\right)}^{3}\left(u_{21}\right)=\left\{a_{1},c_{1},v_{12},v_{22},c_{2},v_{21},a_{4},v_{32}\right\}, \end{array}$$
  $$\begin{array}{c} N_{H^{c}\left(2,m\right)}^{3}\left(a_{i}\right)=\left\{u_{(i-2)1},b_{i-1},u_{(i-2)2},u_{(i-1)2},u_{i2},u_{(i+1)2},b_{i+1},u_{(i+1)1}\right\} \end{array}$$
  for $3\leq i\leq \lfloor \frac{m}{2}\rfloor +1$, and
  $$\begin{array}{c} N_{H^{c}\left(2,m\right)}^{3}\left(u_{i1}\right)=\left\{a_{i-1},v_{(i-2)1},v_{(i-1)2},v_{i2},v_{(i-2)2},v_{i1},v_{(i+1)2},a_{i+2}\right\} \end{array}$$
  for $3\leq i\leq \lceil \frac{m}{2}\rceil$.
  Hence $|N_{H^{c}\left(2,m\right)}^{3}\left(v\right)|=4$ for *v* ∈ *V*_0_, $|N_{H^{c}\left(2,m\right)}^{3}\left(v\right)|=5$ for *v* ∈ *V*_1_, $|N_{H^{c}\left(2,m\right)}^{3}\left(v\right)|=6$ for *v* ∈ *V*_2_, and $|N_{H^{c}\left(2,m\right)}^{3}\left(v\right)|=8$ for any vertex *v* in *V*′ = *V*(*H*^*c*^(2, *m*)) − *V*_0_ − *V*_1_ − *V*_2_. Consequently
  $$\begin{array}{ccc} W_{p}\left(H^{c}\left(2,m\right)\right) & = & \frac{\sum_{v\in V(H^{c}\left(2,m\right))}\left|N_{H^{c}\left(2,m\right)}^{3}\left(v\right)\right|}{2}\\ & = & \frac{4\times |V_{0}\left|+5\times \right|V_{1}\left|+6\times \right|V_{2}\left|+8\times \right|V^{\prime }|}{2}\\ & = & \frac{4\times 6+5\times 6+6\times 6+8\times [6(m+1)-6\times 3]}{2}\\ & = & 24m-3, \end{array}$$
  which also holds for *m* = 1 and *m* = 2.
- If *n* ≥ 3, we have, for *m* = 1 or *m* = 2,
  $$\begin{array}{c} W_{p}\left(H^{c}\left(n,1\right)\right)=9\left(n+1\right) \end{array}$$
  and
  $$\begin{array}{c} W_{p}\left(H^{c}\left(n,2\right)\right)=18\left(n+1\right). \end{array}$$
  When *m* ≥ 3, similar to before we have
  $$\begin{array}{c} |N_{H^{c}\left(n,m\right)}^{3}\left(v\right)|=5\;\;\;\text{for}\;v\in V_{0}, \end{array}$$
  $$\begin{array}{c} |N_{H^{c}\left(n,m\right)}^{3}\left(v\right)|=6\;\;\;\text{for}\;v\in V_{1}, \end{array}$$
  $$\begin{array}{c} |N_{H^{c}\left(n,m\right)}^{3}\left(v\right)|=7\;\;\;\text{for}\;v\in V_{2}, \end{array}$$
  and
  $$\begin{array}{c} |N_{H^{c}\left(n,m\right)}^{3}\left(v\right)|=9\;\;\;\text{for}\;v\in V^{\prime }=V\left(H^{c}\left(n,m\right)\right)-V_{0}-V_{1}-V_{2}. \end{array}$$
  Hence
  $$\begin{array}{ccc} W_{p}\left(H^{c}\left(n,m\right)\right) & = & \frac{\sum_{v\in V(H^{c}\left(n,m\right))}\left|N_{H^{c}\left(n,m\right)}^{3}\left(v\right)\right|}{2}\\ & = & \frac{5\times |V_{0}\left|+6\times \right|V_{1}\left|+7\times \right|V_{2}\left|+9\times \right|V^{\prime }|}{2}\\ & = & \frac{5(2n+2)+6(2n+2)+7(2n+2)+9(2n+2)(m-2)}{2}\\ & = & 9m(n+1), \end{array}$$
  also satisfied by *W*_*p*_(*H*^*c*^(*n*, 1)) and *W*_*p*_(*H*^*c*^(*n*, 2)).

**(3)** Lastly, we will evaluate the Wiener polarity index of *H*^*f*^(*n*, *m*) recursively.

First consider the case *n* = 1. Through direct computation we have, in *H*^*f*^(1, *m*), the followings when *m* ≥ 3:

$|N_{H^{f}\left(1,m\right)}^{3}\left(a_{1}\right)\left|=\right|N_{H^{f}\left(1,m\right)}^{3}\left(b_{m+1}\right)|=2$,

$|N_{H^{f}\left(1,m\right)}^{3}\left(u_{11}\right)\left|=\right|N_{H^{f}\left(1,m\right)}^{3}\left(v_{m1}\right)|=2$,

$|N_{H^{f}\left(1,m\right)}^{3}\left(a_{2}\right)\left|=\right|N_{H^{f}\left(1,m\right)}^{3}\left(b_{m}\right)|=3$,

$|N_{H^{f}\left(1,m\right)}^{3}\left(u_{i1}\right)\left|=\right|N_{H^{f}\left(1,m\right)}^{3}\left(v_{(m+1-i)1}\right)|=4$ for 2 ≤ *i* ≤ *m* − 1,

$|N_{H^{f}\left(1,m\right)}^{3}\left(a_{i}\right)\left|=\right|N_{H^{f}\left(1,m\right)}^{3}\left(b_{m+2-i}\right)|=5$ for 3 ≤ *i* ≤ *m*,

$|N_{H^{f}\left(1,m\right)}^{3}\left(u_{m1}\right)\left|=\right|N_{H^{f}\left(1,m\right)}^{3}\left(v_{11}\right)|=3$,

$|N_{H^{f}\left(1,m\right)}^{3}\left(a_{m+1}\right)\left|=\right|N_{H^{f}\left(1,m\right)}^{3}\left(b_{1}\right)|=4$, and

$|N_{H^{f}\left(1,m\right)}^{3}\left(c_{1}^{*}\right)\left|=\right|N_{H^{f}\left(1,m\right)}^{3}\left(c_{1}\right)|=2$.

Thus

$$\begin{array}{ccc} W_{p}\left(H^{f}\left(1,m\right)\right) & = & \frac{\sum_{v\in V(H^{f}\left(1,m\right))}\left|N_{H^{f}\left(1,m\right)}^{3}\left(v\right)\right|}{2}\\ & = & \frac{[2+2+3+4(m-2)+5(m-2)+3+4+2]\times 2}{2}\\ & = & 9m-2, \end{array}$$

also satisfied by *W*_*p*_(*H*^*f*^(1, 1)) = 7 and *W*_*p*_(*H*^*f*^(1, 2)) = 16.

Now let *n* ≥ 2. The structure of *H*^*f*^(*n*, *m*) yields

$$\begin{array}{ccc} W_{p}\left(H^{f}\left(n,m\right)\right) & = & \left|\{\left(u,v\right)|d_{H^{f}\left(n,m\right)}\left(u,v\right)=3,u,v\in V\left(H^{f}\left(n,m\right)\right)\}\right|\\ & = & |N_{H^{f}\left(n,m\right)}^{3}\left(a_{1}\right)\left|+\right|N_{H^{f}\left(n,m\right)}^{3}\left(u_{11}\right)\left|+\right|N_{H^{f}\left(n,m\right)}^{3}\left(a_{2}\right)|\\ & + & \sum_{i=2}^{m}\left(\right|N_{H^{f}\left(n,m\right)}^{3}\left(u_{i1}\right)|-1)+\sum_{i=3}^{m+1}\left(\right|N_{H^{f}\left(n,m\right)}^{3}\left(a_{i}\right)\left|-1\right)\\ & + & \left(\right|N_{H^{f}\left(n,m\right)}^{3}\left(c_{1}^{*}\right)\left|-1\right)+W_{p}\left(H^{f}\left(n-1,m\right)\right). \end{array}$$

Here $\left(|N_{H^{f}\left(n,m\right)}^{3}\left(u_{i1}\right)|-1\right)$ and $\left(|N_{H^{f}\left(n,m\right)}^{3}\left(a_{i}\right)|-1\right)$ appear because of the double counted terms. By applying this recursion we can explicitly compute *W*_*p*_(*H*^*f*^(*n*, *m*)) for *n* ≥ 2.

For *m* ≥ 3, it is not difficult to check the 3rd neighborhoods individual vertices, we skip the details. As a result we have

$$\begin{array}{ccc} & & |N_{H^{f}\left(n,m\right)}^{3}\left(a_{1}\right)\left|+\right|N_{H^{f}\left(n,m\right)}^{3}\left(u_{11}\right)\left|+\right|N_{H^{f}\left(n,m\right)}^{3}\left(a_{2}\right)|+\sum_{i=2}^{m}\left(\right|N_{H^{f}\left(n,m\right)}^{3}\left(u_{i1}\right)\left|-1\right)\\ & & +\sum_{i=3}^{m+1}\left(\right|N_{H^{f}\left(n,m\right)}^{3}\left(a_{i}\right)\left|-1)+(\right|N_{H^{f}\left(n,m\right)}^{3}\left(c_{1}^{*}\right)\left|-1\right)=9m. \end{array}$$

and

$$\begin{array}{ccc} W_{p}\left(H^{f}\left(n,m\right)\right) & = & 9m+W_{p}\left(H^{f}\left(n-1,m\right)\right)\\ & = & 9m+9m+W_{p}\left(H^{f}\left(n-2,m\right)\right)\\ & = & \cdots \cdots \\ & = & 9m\times \left(n-1\right)+W_{p}\left(H^{f}\left(1,m\right)\right)\\ & = & 9nm-2 \end{array}$$

for *m* ≥ 3. It is easy to check *W*_*p*_(*H*^*f*^(*n*, 1)) = 9*n* − 2 and *W*_*p*_(*H*^*f*^(*n*, 2)) = 18*n* − 2, and hence *W*_*p*_(*H*^*f*^(*n*, *m*)) = 9*nm* − 2 for *n* ≥ 2 and *m* ≥ 1. Furthermore, we have *W*_*p*_(*H*^*f*^(1, *m*)) = 9*m* − 2. Thus we conclude that *W*_*p*_(*H*^*f*^(*n*, *m*)) = 9*nm* − 2 for *n* ≥ 1 and *m* ≥ 1.

**Remark 3** *For small values of* *n* *and* *m*, *direct computation yields*

*W*_*p*_(*H*^*t*^(1, 1)) = 4, *W*_*p*_(*H*^*t*^(1, 2)) = *W*_*p*_(*H*^*t*^(2, 1)) = 18, *W*_*p*_(*H*^*t*^(2, 2)) = 54.

## The Wiener polarity index of the triangular lattices

**Fig 3:**
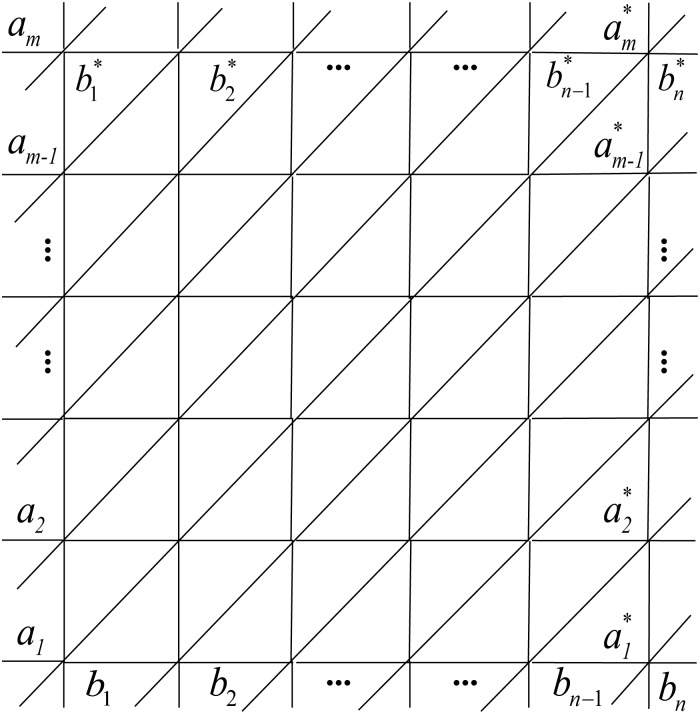
The triangular lattice.

We now turn our attention to the Wiener polarity index of the triangular lattices. Again our notations follow [50]. The triangular lattices with toroidal, cylindrical and free boundary conditions are respectively denoted by *T*^*t*^(*n*, *m*), *T*^*c*^(*n*, *m*) and *T*^*f*^(*n*, *m*). It is not hard to see, that the triangular lattice with toroidal boundary condition *T*^*t*^(*n*, *m*) can be considered as an *n* × *m* square lattice *C*_*n*_□*C*_*m*_ with toroidal boundary condition with an additional diagonal edge added to every square. As in Fig 3, $\left(a_{1},a_{1}^{*}\right)$, $\left(a_{2},a_{2}^{*}\right)$, …, $\left(a_{m},a_{m}^{*}\right)$; $\left(b_{1},b_{1}^{*}\right)$, $\left(b_{2},b_{2}^{*}\right)$, …, $\left(b_{n},b_{n}^{*}\right)$; $\left(b_{2},b_{1}^{*}\right)$, $\left(b_{3},b_{2}^{*}\right)$, …, $\left(b_{n},b_{n-1}^{*}\right)$, $\left(b_{1},b_{n}^{*}\right)=\left(a_{1},a_{m}^{*}\right)$; $\left(a_{2},a_{1}^{*}\right)$, $\left(a_{3},a_{2}^{*}\right)$, …, $\left(a_{m},a_{m-1}^{*}\right)$ are edges. Note that *a*_1_ = *b*_1_, $a_{1}^{*}=b_{n}$, $a_{m}=b_{1}^{*}$ and $a_{m}^{*}=b_{n}^{*}$. The triangular lattice with cylindrical boundary condition *T*^*c*^(*n*, *m*) is obtained by deleting the edges $\left(b_{1},b_{1}^{*}\right)$, $\left(b_{2},b_{2}^{*}\right)$, …, $\left(b_{n},b_{n}^{*}\right)$; $\left(b_{2},b_{1}^{*}\right)$, $\left(b_{3},b_{2}^{*}\right)$, …, $\left(b_{n},b_{n-1}^{*}\right)$, $\left(b_{1},b_{n}^{*}\right)$ from *T*^*t*^(*n*, *m*). If we further remove the edges $\left(a_{1},a_{1}^{*}\right)$, $\left(a_{2},a_{2}^{*}\right)$, …, $\left(a_{m},a_{m}^{*}\right)$; $\left(a_{2},a_{1}^{*}\right)$, $\left(a_{3},a_{2}^{*}\right)$, …, $\left(a_{m},a_{m-1}^{*}\right)$ from *T*^*c*^(*n*, *m*), the triangular lattice with free boundary condition *T*^*f*^(*n*, *m*) is then obtained. Since it has been established that *C*_*n*_□*C*_*m*_ exists for *n* ≥ 3 and *m* ≥ 3, in what follows we assume *n* ≥ 3 and *m* ≥ 3 for all the triangular lattices considered.

**Theorem 3** *Let* *T*^*f*^(*n*, *m*), *T*^*c*^(*n*, *m*) *and* *T*^*t*^(*n*, *m*) *be the triangular lattices with free, cylindrical and toroidal boundary conditions respectively. Then we have*

(i) *For* *n* ≥ 3 *and* *m* ≥ 3, *W*_*p*_(*T*^*f*^(*n*, *m*)) = 9*nm* − 18*n* − 18*m* + 31;

(ii)

$$\begin{array}{c} W_{p}\left(T^{c}\left(n,m\right)\right)=\{\begin{array}{cc} 9m-27 & \text{if}\;n=3\text{,}\;m\geq 3\text{;}\\ 20m-56 & \text{if}\;n=4\text{,}\;m\geq 3\text{;}\\ 35m-85 & \text{if}\;n=5\text{,}\;m\geq 3\text{;}\\ 51m-108 & \text{if}\;n=6\text{,}\;m\geq 3\text{;}\\ 9nm-18n & \text{if}\;n\geq 7\text{,}\;m\geq 3; \end{array} \end{array}$$

(iii)

$$\begin{array}{c} W_{p}\left(T^{t}\left(n,m\right)\right)=\{\begin{array}{cc} 9m & \text{if}\;n=3\text{,}\;m\geq 7\text{;}\\ 20m & \text{if}\;n=4\text{,}\;m\geq 7\text{;}\\ 35m & \text{if}\;n=5\text{,}\;m\geq 7\text{;}\\ 51m & \text{if}\;n=6\text{,}\;m\geq 7\text{;}\\ 9nm & \text{if}\;n\geq 7\text{,}\;m\geq 7. \end{array} \end{array}$$

*Moreover, for small values of* *n* *and* *m* *we have the followings*:

*W*_*p*_(*T*^*t*^(*n*, *m*)) = 0 if *n* = 3, 3 ≤ *m* ≤ 5 or *n* = *m* = 4;

*W*_*p*_(*T*^*t*^(3, 6)) = 27; *W*_*p*_(*T*^*t*^(4, 5)) = 20; *W*_*p*_(*T*^*t*^(4, 6)) = 72;

*W*_*p*_(*T*^*t*^(5, 5)) = 75; *W*_*p*_(*T*^*t*^(5, 6)) = 165; *W*_*p*_(*T*^*t*^(6, 6)) = 270.

**Fig 4:**
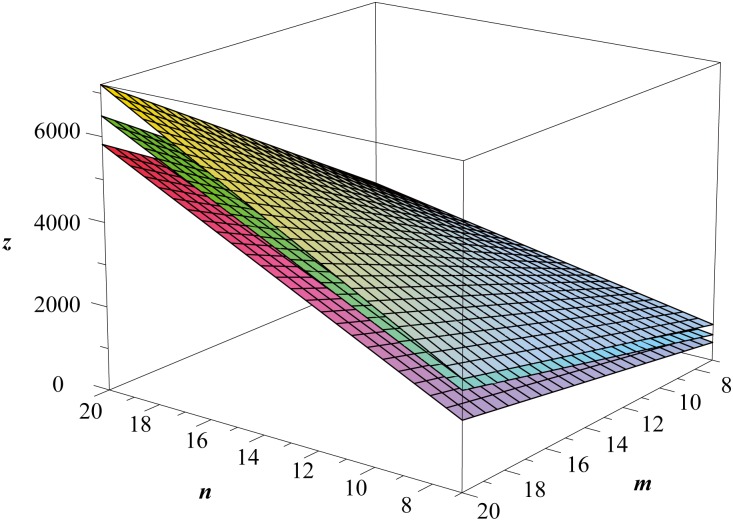
The Wiener polarity index of *T*^*t*^(*n*, *m*), *T*^*c*^(*n*, *m*) and *T*^*f*^(*n*, *m*).

**Remark 4** *As illustrated in* Fig 4, *we generally have*

$$\begin{array}{c} W_{p}\left(T^{t}\left(n,m\right)\right)>W_{p}\left(T^{c}\left(n,m\right)\right)>W_{p}\left(T^{f}\left(n,m\right)\right), \end{array}$$

*with the common asymptotic value* 9*mn* *as both* *m* *an* *n* *approaches infinity. It is interesting to note that the Wiener polarity index of the triangular lattices and that of the hexagonal lattices are approximately the same*.

*Proof.* We consider each of the three statements.

**(i)** First we consider the triangular lattice with free boundary condition, *T*^*f*^(*n*, *m*), with the assumption *n* ≥ *m*.

**Case 1** When *m* = 3:

- If *n* = 3, *W*_*p*_(*T*^*f*^(3, 3)) = 4.
- If *n* ≥ 4, we have
  $$\begin{array}{c} |N_{T^{f}\left(n,3\right)}^{3}\left(a_{1}\right)\left|=3;\;\;\right|N_{T^{f}\left(n,3\right)}^{3}\left(a_{2}\right)\left|=3;\;\;\right|N_{T^{f}\left(n,3\right)}^{3}\left(a_{3}\right)|=3. \end{array}$$
  Thus
  $$\begin{array}{ccc} W_{p}\left(T^{f}\left(n,3\right)\right) & = & \sum_{i=1}^{3}\left|N_{T^{f}\left(n,3\right)}^{3}\left(a_{i}\right)\right|+W_{p}\left(T^{f}\left(n-1,3\right)\right)\\ & = & 9+W_{p}\left(T^{f}\left(n-1,3\right)\right)\\ & = & 9+\sum_{i=1}^{3}\left|N_{T^{f}\left(n-1,3\right)}^{3}\left(a_{i}\right)\right|+W_{p}\left(T^{f}\left(n-2,3\right)\right)\\ & = & 9+9+W_{p}\left(T^{f}\left(n-2,3\right)\right)\\ & = & \cdots \\ & = & 9\left(n-3\right)+W_{p}\left(T^{f}\left(3,3\right)\right)=9n-23, \end{array}$$
  also satisfied by *W*_*p*_(*T*^*f*^(3, 3)) = 4.

**Case 2** When *m* = 4, for *n* ≥ *m* we have

$$\begin{array}{c} |N_{T^{f}\left(n,4\right)}^{3}\left(a_{1}\right)\left|=7;\;\right|N_{T^{f}\left(n,4\right)}^{3}\left(a_{2}\right)\left|=4;\;\right|N_{T^{f}\left(n,4\right)}^{3}\left(a_{3}\right)\left|=4;\;\right|N_{T^{f}\left(n,4\right)}^{3}\left(a_{4}\right)|=4. \end{array}$$

Hence

$$\begin{array}{ccc} W_{p}\left(T^{f}\left(n,4\right)\right) & = & \sum_{i=1}^{4}\left|N_{T^{f}\left(n,4\right)}^{3}\left(a_{i}\right)\right|-W_{p}\left(P_{4}\right)+W_{p}\left(T^{f}\left(n-1,4\right)\right)\\ & = & \left(19-1\right)+W_{p}\left(T^{f}\left(n-1,4\right)\right)\\ & = & 18+\sum_{i=1}^{4}\left|N_{T^{f}\left(n-1,4\right)}^{3}\left(a_{i}\right)\right|-W_{p}\left(P_{4}\right)+W_{p}\left(T^{f}\left(n-2,4\right)\right)\\ & = & 18+18+W_{p}\left(T^{f}\left(n-2,4\right)\right)\\ & = & \cdots \\ & = & 18\left(n-3\right)+W_{p}\left(T^{f}\left(3,4\right)\right)\\ & = & 18\left(n-3\right)+W_{p}\left(T^{f}\left(4,3\right)\right)\\ & = & 18n-41. \end{array}$$

**Case 3** When *m* = 5, similar to Case 2 we have *W*_*p*_(*T*^*f*^(*n*, 5)) = 27*n* − 59 for *n* ≥ *m* = 5.

**Case 4** When *m* ≥ 6 and *n* ≥ *m*. Simple computation yields

$$\begin{array}{c} |N_{T^{f}\left(n,m\right)}^{3}\left(a_{1}\right)\left|=7;\;\right|N_{T^{f}\left(n,m\right)}^{3}\left(a_{2}\right)\left|=8;\;\right|N_{T^{f}\left(n,m\right)}^{3}\left(a_{3}\right)|=9; \end{array}$$

$$\begin{array}{c} |N_{T^{f}\left(n,m\right)}^{3}\left(a_{4}\right)\left|=\cdots =\right|N_{T^{f}\left(n,m\right)}^{3}\left(a_{m-3}\right)|=10; \end{array}$$

$$\begin{array}{c} |N_{T^{f}\left(n,m\right)}^{3}\left(a_{m-2}\right)\left|=6;\;\right|N_{T^{f}\left(n,m\right)}^{3}\left(a_{m-1}\right)\left|=5;\;\right|N_{T^{f}\left(n,m\right)}^{3}\left(a_{m}\right)|=4. \end{array}$$

Noting that *W*_*p*_(*P*_*m*_) = *m* − 3 and *W*_*p*_(*T*^*f*^(3, *m*)) = *W*_*p*_(*T*^*f*^(*m*, 3)) = 9*m* − 23, we have

$$\begin{array}{ccc} W_{p}\left(T^{f}\left(n,m\right)\right) & = & \sum_{i=1}^{m}\left|N_{T^{f}\left(n,m\right)}^{3}\left(a_{i}\right)\right|-W_{p}\left(P_{m}\right)+W_{p}\left(T^{f}\left(n-1,m\right)\right)\\ & = & \left(10m-21\right)-\left(m-3\right)+W_{p}\left(T^{f}\left(n-1,m\right)\right)\\ & = & \left(9m-18\right)+\sum_{i=1}^{m}\left|N_{T^{f}\left(n-1,m\right)}^{3}\left(a_{i}\right)\right|-W_{p}\left(P_{m}\right)\\ & & +W_{p}\left(T^{f}\left(n-2,m\right)\right)\\ & = & \left(9m-18\right)+\left(9m-18\right)+W_{p}\left(T^{f}\left(n-2,m\right)\right)\\ & = & \cdots \\ & = & \left(9m-18\right)\times \left(n-3\right)+W_{p}\left(T^{f}\left(3,m\right)\right)\\ & = & 9nm-18n-18m+31. \end{array}$$

Since this formula coincides with our findings for smaller values of *m*, we conclude that *W*_*p*_(*T*^*f*^(*n*, *m*)) = 9*nm* − 18*n* − 18*m* + 31 for *n* ≥ 3 and *m* ≥ 3.

**(ii)** Next we consider the triangular lattice with cylindrical boundary condition *T*^*c*^(*n*, *m*). The symmetric structure of *T*^*c*^(*n*, *m*) indicates that the vertices from the same row have the same number of 3rd neighbors. Thus it suffices to compute $N_{T^{c}\left(n,m\right)}^{3}\left(a_{i}\right)$ for *i* = 1, 2, …, *m* to obtain *W*_*p*_(*T*^*c*^(*n*, *m*)).

**Case 1** If *n* = 3; first suppose *m* ≥ 7. Direct computation yields

$$\begin{array}{c} |N_{T^{c}\left(3,m\right)}^{3}\left(a_{i}\right)|=3\;\;\text{for}\;i=1\text{,}\;\text{2,}\;\text{3,}\;m-2\text{,}\;m-1\text{,}\;m \end{array}$$

and

$$\begin{array}{c} |N_{T^{c}\left(3,m\right)}^{3}\left(a_{i}\right)|=6\;\;\text{for}\;4\leq i\leq m-3. \end{array}$$

Thus Lemma 4 implies that

$$\begin{array}{ccc} W_{p}\left(T^{c}\left(3,m\right)\right) & = & \frac{\sum_{v\in V(T^{c}\left(3,m\right))}\left|N_{T^{c}\left(3,m\right)}^{3}\left(v\right)\right|}{2}\\ & = & \frac{(\sum_{i=1}^{m}\left|N_{T^{c}\left(3,m\right)}^{3}\left(a_{i}\right)\right|)\times 3}{2}\\ & = & 9m-27. \end{array}$$

On the other hand, for 3 ≤ *m* ≤ 6, one can verify *W*_*p*_(*T*^*c*^(3, *m*)) = 9*m* − 27 through direct computation.

**Case 2** If *n* = 4, 5, or 6; Following essentially the same arguments as that of Case 1, we have

$$\begin{array}{c} W_{p}\left(T^{c}\left(4,m\right)\right)=20m-56\;\;\;for\;m\geq 3; \end{array}$$

$$\begin{array}{c} W_{p}\left(T^{c}\left(5,m\right)\right)=35m-85\;\;\;for\;m\geq 3; \end{array}$$

$$\begin{array}{c} W_{p}\left(T^{c}\left(6,m\right)\right)=51m-108\;\;\;for\;m\geq 3. \end{array}$$

**Case 3** If *n* ≥ 7:

- When *m* = 3, it is easy to see that $|N_{T^{c}\left(n,3\right)}^{3}\left(a_{i}\right)|=6$ for *i* = 1, 2, 3, and hence Lemma 4 implies
  $$\begin{array}{ccc} W_{p}\left(T^{c}\left(n,3\right)\right) & = & \frac{\sum_{v\in V(T^{c}\left(n,3\right))}\left|N_{T^{c}\left(n,3\right)}^{3}\left(v\right)\right|}{2}\\ & = & \frac{(\sum_{i=1}^{3}\left|N_{T^{c}\left(n,3\right)}^{3}\left(a_{i}\right)\right|)\times n}{2}\\ & = & 9n. \end{array}$$
- When *m* = 4, we have $|N_{T^{c}\left(n,4\right)}^{3}\left(a_{1}\right)\left|=\right|N_{T^{c}\left(n,4\right)}^{3}\left(a_{4}\right)|=10$ and $|N_{T^{c}\left(n,4\right)}^{3}\left(a_{2}\right)\left|=\right|N_{T^{c}\left(n,4\right)}^{3}\left(a_{3}\right)|=8$. Thus
  $$\begin{array}{ccc} W_{p}\left(T^{c}\left(n,4\right)\right) & = & \frac{\sum_{v\in V(T^{c}\left(n,4\right))}\left|N_{T^{c}\left(n,4\right)}^{3}\left(v\right)\right|}{2}\\ & = & \frac{(\sum_{i=1}^{4}\left|N_{T^{c}\left(n,4\right)}^{3}\left(a_{i}\right)\right|)\times n}{2}\\ & = & 18n. \end{array}$$
- Similarly, when *m* = 5 or 6 we have
  $$\begin{array}{c} W_{p}\left(T^{c}\left(n,5\right)\right)=27n; \end{array}$$
  $$\begin{array}{c} W_{p}\left(T^{c}\left(n,6\right)\right)=36n. \end{array}$$
- When *m* ≥ 7, examining the 3rd neighborhoods yields
  $$\begin{array}{c} |N_{T^{c}\left(n,m\right)}^{3}\left(a_{1}\right)\left|=\right|N_{T^{c}\left(n,m\right)}^{3}\left(a_{m}\right)|=10; \end{array}$$
  $$\begin{array}{c} |N_{T^{c}\left(n,m\right)}^{3}\left(a_{2}\right)\left|=\right|N_{T^{c}\left(n,m\right)}^{3}\left(a_{m-1}\right)|=12; \end{array}$$
  $$\begin{array}{c} |N_{T^{c}\left(n,m\right)}^{3}\left(a_{3}\right)\left|=\right|N_{T^{c}\left(n,m\right)}^{3}\left(a_{m-2}\right)|=14; \end{array}$$
  $$\begin{array}{c} |N_{T^{c}\left(n,m\right)}^{3}\left(a_{i}\right)|=18\;\;for\;4\leq i\leq m-3. \end{array}$$
  Consequently
  $$\begin{array}{ccc} W_{p}\left(T^{c}\left(n,m\right)\right) & = & \frac{\sum_{v\in V(T^{c}\left(n,m\right))}\left|N_{T^{c}\left(n,m\right)}^{3}\left(v\right)\right|}{2}\\ & = & \frac{(\sum_{i=1}^{m}\left|N_{T^{c}\left(n,m\right)}^{3}\left(a_{i}\right)\right|)\times n}{2}\\ & = & 9nm-18n. \end{array}$$
  Again this formula can be verified with *m* = 3, 4, 5, 6, and hence we may conclude that *W*_*p*_(*T*^*c*^(*n*, *m*)) = 9*nm* − 18*n* for *n* ≥ 7 and *m* ≥ 3.

**(iii)** Lastly, we consider *T*^*t*^(*n*, *m*) with the assumption that *m* ≥ *n*. It is easy to see that all vertices share the same number of 3rd neighbors.

For small values of *n* and *m*, we have *W*_*p*_(*T*^*t*^(*n*, *m*)) = 0 for *n* = 3 and 3 ≤ *m* ≤ 5 or *n* = *m* = 4; *W*_*p*_(*T*^*t*^(3, 6)) = 27; *W*_*p*_(*T*^*t*^(4, 5)) = 20; *W*_*p*_(*T*^*t*^(4, 6)) = 72; *W*_*p*_(*T*^*t*^(5, 5)) = 75; *W*_*p*_(*T*^*t*^(5, 6)) = 165; *W*_*p*_(*T*^*t*^(6, 6)) = 270.

For *m* ≥ 7 we consider different cases depending on the value of *n*.

- If *n* = 3 and *m* ≥ 7, then $|N_{T^{t}\left(3,m\right)}^{3}\left(v\right)|=6$ for any vertex *v* ∈ *V*(*T*^*t*^(3, *m*)) and
  $$\begin{array}{ccc} W_{p}\left(T^{t}\left(3,m\right)\right) & = & \frac{\sum_{v\in V(T^{t}\left(3,m\right))}\left|N_{T^{t}\left(3,m\right)}^{3}\left(v\right)\right|}{2}\\ & = & \frac{6\times 3m}{2}\\ & = & 9m. \end{array}$$
- If *n* = 4 and *m* ≥ 7, $|N_{T^{t}\left(4,m\right)}^{3}\left(v\right)|=10$ for any vertex *v* ∈ *V*(*T*^*t*^(4, *m*)) and *W*_*p*_(*T*^*t*^(4, *m*)) = 20*m*.
- If *n* = 5 and *m* ≥ 7, $|N_{T^{t}\left(5,m\right)}^{3}\left(v\right)|=14$ for any vertex *v* ∈ *V*(*T*^*t*^(5, *m*)) and *W*_*p*_(*T*^*t*^(5, *m*)) = 35*m*.
- If *n* = 6 and *m* ≥ 7, $|N_{T^{t}\left(6,m\right)}^{3}\left(v\right)|=17$ for any vertex *v* ∈ *V*(*T*^*t*^(6, *m*)) and *W*_*p*_(*T*^*t*^(6, *m*)) = 51*m*.
- If *n* ≥ 7 and *m* ≥ 7, direct computation shows $|N_{T^{t}\left(n,m\right)}^{3}\left(v\right)|=18$ for any vertex *v* ∈ *V*(*T*^*t*^(*n*, *m*)). Hence
  $$\begin{array}{ccc} W_{p}\left(T^{t}\left(n,m\right)\right) & = & \frac{\sum_{v\in V(T^{t}\left(n,m\right))}\left|N_{T^{t}\left(n,m\right)}^{3}\left(v\right)\right|}{2}\\ & = & \frac{18\times nm}{2}\\ & = & 9nm. \end{array}$$

## The Wiener polarity index of the 3^3^ ⋅ 4^2^ lattices

**Fig 5:**
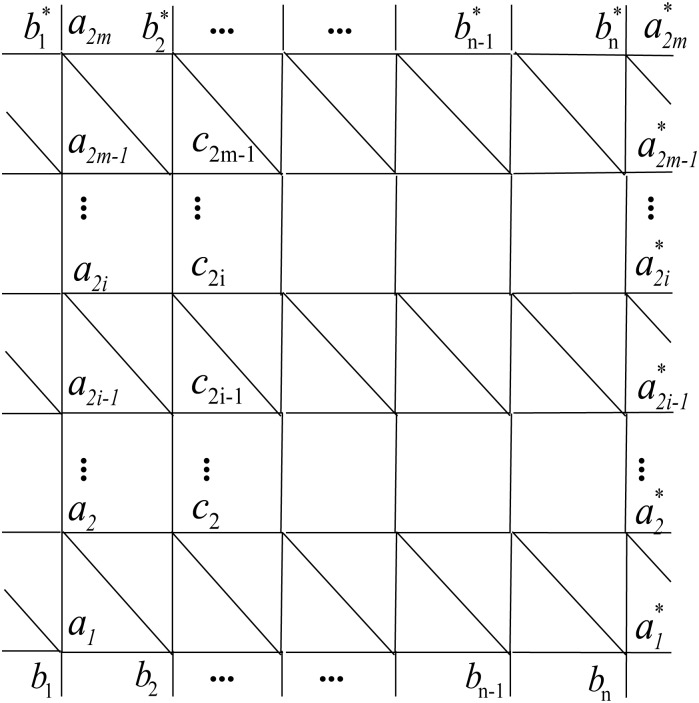
The 3^3^ ⋅ 4^2^ lattice.

We conclude our study by considering the Wiener polarity index of the 3^3^ ⋅ 4^2^ lattices, following the notations of [50]. The 3^3^ ⋅ 4^2^ lattice with toroidal boundary condition, denoted by *S*^*t*^(*n*, 2*m*), can be constructed from the square lattice *C*_2*m*_□*C*_*n*_ by adding a diagonal edge in each square of every other row, as shown in Fig 5. Here *a*_1_ = *b*_1_, $a_{2m}=b_{1}^{*}$, $a_{1}^{*}=b_{n}$, $a_{2m}^{*}=b_{n}^{*}$, and $\left(a_{1},a_{1}^{*}\right)$, $\left(a_{2},a_{2}^{*}\right)$, …, $\left(a_{2m},a_{2m}^{*}\right)$; $\left(b_{1},b_{1}^{*}\right)$, $\left(b_{2},b_{2}^{*}\right)$, …, $\left(b_{n},b_{n}^{*}\right)$; $\left(a_{1},a_{2}^{*}\right)$, $\left(a_{3},a_{4}^{*}\right)$, …, $\left(a_{2m-1},a_{2m}^{*}\right)$ are edges. If we remove the edges $\left(b_{1},b_{1}^{*}\right)$, $\left(b_{2},b_{2}^{*}\right)$, …, $\left(b_{n},b_{n}^{*}\right)$ of *S*^*t*^(*n*, 2*m*), then the 3^3^ ⋅ 4^2^ lattice with cylindrical boundary condition, denoted by *S*^*c*^(*n*, 2*m*), is obtained. The 3^3^ ⋅ 4^2^ lattice *S*^*f*^(*n*, 2*m*) with free boundary condition is obtained by further removing edges $\left(a_{1},a_{1}^{*}\right)$, $\left(a_{2},a_{2}^{*}\right)$, …, $\left(a_{2m},a_{2m}^{*}\right)$; $\left(a_{1},a_{2}^{*}\right)$, $\left(a_{3},a_{4}^{*}\right)$, …, $\left(a_{2m-1},a_{2m}^{*}\right)$ from *S*^*c*^(*n*, 2*m*). Similar to before, we will assume *n* ≥ 3 and *m* ≥ 2 when the 3^3^ ⋅ 4^2^ lattices are discussed.

**Theorem 4** *Let* *S*^*f*^(*n*, 2*m*), *S*^*c*^(*n*, 2*m*) *and* *S*^*t*^(*n*, 2*m*) *be the* 3^3^ ⋅ 4^2^ *lattices with free, cylindrical and toroidal boundary conditions, respectively. Then*

(i) *For* *n* ≥ 3 *and* *m* ≥ 2, *W*_*p*_(*S*^*f*^(*n*, 2*m*)) = 15*nm* − 13*n* − 25*m* + 15;

(ii)

$$\begin{array}{c} W_{p}\left(S^{c}\left(n,2m\right)\right)=\{\begin{array}{cc} 21m-27 & \text{if}\;n=3\text{,}\;m\geq 2\text{;}\\ 40m-48 & \text{if}\;n=4\text{,}\;m\geq 2\text{;}\\ 60m-65 & \text{if}\;n=5\text{,}\;m\geq 2\text{;}\\ 84m-78 & \text{if}\;n=6\text{,}\;m\geq 2\text{;}\\ 15nm-13n & \text{if}\;n\geq 7\text{,}\;m\geq 2\text{;} \end{array} \end{array}$$

(iii)

$$\begin{array}{c} W_{p}\left(S^{t}\left(n,2m\right)\right)=\{\begin{array}{cc} 21m & \text{if}\;n=3\text{,}\;m\geq 4\text{;}\\ 40m & \text{if}\;n=4\text{,}\;m\geq 4\text{;}\\ 60m & \text{if}\;n=5\text{,}\;m\geq 4\text{;}\\ 84m & \text{if}\;n=6\text{,}\;m\geq 4\text{;}\\ 16n & \text{if}\;n\geq 7\text{,}\;m=2\text{;}\\ 42n & \text{if}\;n\geq 7\text{,}\;m=3\text{;}\\ 15nm & \text{if}\;n\geq 7\text{,}\;m\geq 4\text{.} \end{array} \end{array}$$

*In addition, for small* *n* *and* *m* *we have* *W*_*p*_(*S*^*t*^(3, 4)) = 0; *W*_*p*_(*S*^*t*^(3, 6)) = 45; *W*_*p*_(*S*^*t*^(4, 4)) = 16; *W*_*p*_(*S*^*t*^(4, 6)) = 108; *W*_*p*_(*S*^*t*^(5, 4)) = 50; *W*_*p*_(*S*^*t*^(5, 6)) = 165; *W*_*p*_(*S*^*t*^(6, 4)) = 84; *W*_*p*_(*S*^*t*^(6, 6)) = 234.

**Fig 6:**
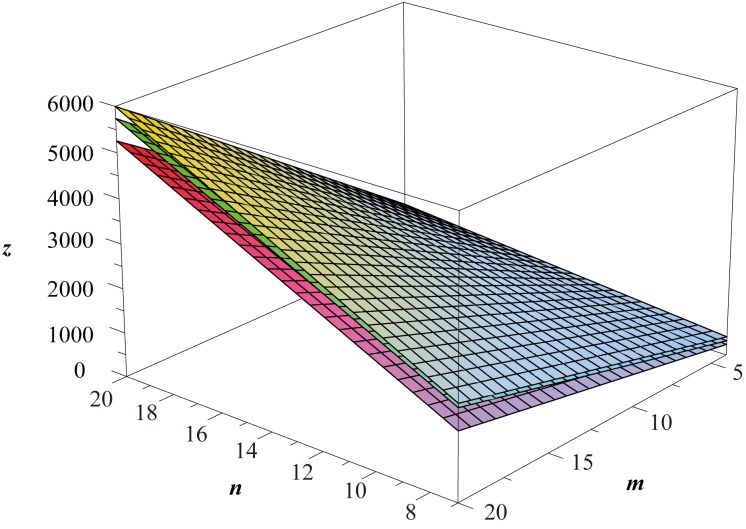
The Wiener polarity index of *S*^*t*^(*n*, *m*), *S*^*c*^(*n*, *m*) and *S*^*f*^(*n*, *m*).

**Remark 5** *As illurstrated in* Fig 6, *we generally have*

$$\begin{array}{c} W_{p}\left(S^{t}\left(n,m\right)\right)>W_{p}\left(S^{c}\left(n,m\right)\right)>W_{p}\left(S^{f}\left(n,m\right)\right), \end{array}$$

*with the common asymptotic value* 15*mn* *as both* *m* *an* *n* *approaches infinity*.

*Proof.* We consider each of the three cases as follows:

**(i)** We start with the 3^3^ ⋅ 4^2^ lattice *S*^*f*^(*n*, 2*m*) with free boundary condition. First we consider *W*_*p*_(*S*^*f*^(*n*, 2*m*)), noting that $|N_{S^{f}\left(3,2m\right)}^{3}\left(a_{i}\right)\left|=\right|N_{S^{f}\left(3,2m\right)}^{3}\left(a_{2m+1-i}^{*}\right)|$ for 1 ≤ *i* ≤ 2*m*, thus for *m* ≥ 4 we have

$$\begin{array}{c} |N_{S^{f}\left(3,2m\right)}^{3}\left(a_{1}\right)\left|=\right|N_{S^{f}\left(3,2m\right)}^{3}\left(a_{2m}^{*}\right)\left|=3;\;\right|N_{S^{f}\left(3,2m\right)}^{3}\left(a_{2}\right)\left|=\right|N_{S^{f}\left(3,2m\right)}^{3}\left(a_{2m-1}^{*}\right)|=3; \end{array}$$

$$\begin{array}{c} |N_{S^{f}\left(3,2m\right)}^{3}\left(a_{3}\right)\left|=\right|N_{S^{f}\left(3,2m\right)}^{3}\left(a_{2m-2}^{*}\right)\left|=5;\;\right|N_{S^{f}\left(3,2m\right)}^{3}\left(a_{2m-2}\right)\left|=\right|N_{S^{f}\left(3,2m\right)}^{3}\left(a_{3}^{*}\right)|=6; \end{array}$$

$$\begin{array}{c} |N_{S^{f}\left(3,2m\right)}^{3}\left(a_{2m-1}\right)\left|=\right|N_{S^{f}\left(3,2m\right)}^{3}\left(a_{2}^{*}\right)\left|=5;\;\right|N_{S^{f}\left(3,2m\right)}^{3}\left(a_{2m}\right)\left|=\right|N_{S^{f}\left(3,2m\right)}^{3}\left(a_{1}^{*}\right)|=4; \end{array}$$

$$\begin{array}{c} |N_{S^{f}\left(3,2m\right)}^{3}\left(a_{i}\right)\left|=\right|N_{S^{f}\left(3,2m\right)}^{3}\left(a_{2m+1-i}^{*}\right)|=7\;\;\text{for}\;4\leq i\leq 2m-3; \end{array}$$

$$\begin{array}{c} |N_{S^{f}\left(3,2m\right)}^{3}\left(b_{2}\right)\left|=3;\;\right|N_{S^{f}\left(3,2m\right)}^{3}\left(c_{2}\right)\left|=3;\;\right|N_{S^{f}\left(3,2m\right)}^{3}\left(c_{3}\right)|=4; \end{array}$$

$$\begin{array}{c} |N_{S^{f}\left(3,2m\right)}^{3}\left(c_{2m-2}\right)\left|=4;\;\right|N_{S^{f}\left(3,2m\right)}^{3}\left(c_{2m-1}\right)\left|=3;\;\right|N_{S^{f}\left(3,2m\right)}^{3}\left(b_{2}^{*}\right)|=3; \end{array}$$

$$\begin{array}{c} |N_{S^{f}\left(3,2m\right)}^{3}\left(c_{i}\right)|=6\;\;\text{for}\;4\leq i\leq 2m-3. \end{array}$$

Plugging into Eq (2), we have *W*_*p*_(*S*^*f*^(3, 2*m*)) = 20*m* − 24 for *m* ≥ 4. Since *W*_*p*_(*S*^*f*^(3, 4)) = 16 = 20 × 2 − 24 when *m* = 2 and *W*_*p*_(*S*^*f*^(3, 6)) = 36 = 20 × 3 − 24 when *m* = 3, we conclude that *W*_*p*_(*S*^*f*^(3, 2*m*)) = 20*m* − 24 for *m* ≥ 2.

Now suppose *n* ≥ 4. Observe that if the vertices in the first column are removed from *S*^*f*^(*n*, 2*m*), then *S*^*f*^(*n* − 1, 2*m*) is obtained. We now consider the vertices in the first column of *S*^*f*^(*n*, 2*m*) and their 3rd neighbors:

$$\begin{array}{c} |N_{S^{f}\left(n,2m\right)}^{3}\left(a_{1}\right)\left|=4;\;\right|N_{S^{f}\left(n,2m\right)}^{3}\left(a_{2}\right)\left|=5;\;\right|N_{S^{f}\left(n,2m\right)}^{3}\left(a_{3}\right)|=6; \end{array}$$

$$\begin{array}{c} |N_{S^{f}\left(n,2m\right)}^{3}\left(a_{i}\right)|=9\;\;\;\text{for}\;i=4,6,\ldots ,2m-4; \end{array}$$

$$\begin{array}{c} |N_{S^{f}\left(n,2m\right)}^{3}\left(a_{i}\right)|=8\;\;\;\text{for}\;i=5,7,\ldots ,2m-3; \end{array}$$

$$\begin{array}{c} |N_{S^{f}\left(n,2m\right)}^{3}\left(a_{2m-2}\right)\left|=8;\;\right|N_{S^{f}\left(n,2m\right)}^{3}\left(a_{2m-1}\right)\left|=6;\;\right|N_{S^{f}\left(n,2m\right)}^{3}\left(a_{2m}\right)|=6; \end{array}$$

Applying Lemma 4 together with the fact that *W*_*p*_(*P*_*n*_) = *n* − 3, have, for *m* ≥ 4,

$$\begin{array}{ccc} W_{p}\left(S^{f}\left(n,2m\right)\right) & = & \sum_{i=1}^{2m}\left|N_{S^{f}\left(n,2m\right)}^{3}\left(a_{i}\right)\right|-W_{p}\left(P_{2m}\right)+W_{p}\left(S^{f}\left(n-1,2m\right)\right)\\ & = & \left(15m-13\right)+W_{p}\left(S^{f}\left(n-1,2m\right)\right)\\ & = & \left(15m-13\right)+\sum_{i=1}^{2m}\left|N_{S^{f}\left(n-1,2m\right)}^{3}\left(a_{i}\right)\right|-W_{p}\left(P_{2m}\right)\\ & & +W_{p}\left(S^{f}\left(n-2,2m\right)\right)\\ & = & \left(15m-13\right)+\left(15m-13\right)+W_{p}\left(T^{f}\left(n-2,m\right)\right)\\ & = & \cdots \\ & = & \left(15m-13\right)\times \left(n-3\right)+W_{p}\left(S^{f}\left(3,2m\right)\right)\\ & = & 15nm-13n-25m+15. \end{array}$$

Again this formula can be verified for small values of *n* and *m*. Hence *W*_*p*_(*S*^*f*^(*n*, 2*m*)) = 15*nm* − 13*n* − 25*m* + 15 for *m* ≥ 2.

**(ii)** Next we consider the 3^3^ ⋅ 4^2^ lattice *S*^*c*^(*n*, 2*m*) with cylindrical boundary condition. In this case the vertices of *S*^*c*^(*n*, 2*m*) in the same row have the same number of 3rd neighbors. Hence it is sufficient to compute $|N_{S^{c}\left(n,2m\right)}^{3}\left(a_{i}\right)|$ for *i* = 1, 2, …, 2*m*.

**Case 1** If *n* = 3 and *m* ≥ 2; When *m* ≥ 4 we have

$$\begin{array}{c} |N_{S^{c}\left(3,2m\right)}^{3}\left(a_{1}\right)\left|=\right|N_{S^{c}\left(3,2m\right)}^{3}\left(a_{2m}\right)|=4; \end{array}$$

$$\begin{array}{c} |N_{S^{c}\left(3,2m\right)}^{3}\left(a_{2}\right)\left|=\right|N_{S^{c}\left(3,2m\right)}^{3}\left(a_{2m-1}\right)|=3; \end{array}$$

$$\begin{array}{c} |N_{S^{c}\left(3,2m\right)}^{3}\left(a_{3}\right)\left|=\right|N_{S^{c}\left(3,2m\right)}^{3}\left(a_{2m-2}\right)|=5; \end{array}$$

$$\begin{array}{c} |N_{S^{c}\left(3,2m\right)}^{3}\left(a_{i}\right)|=7\;\;\text{for}\;4\leq i\leq 2m-3. \end{array}$$

Then Lemma 4 implies

$$\begin{array}{ccc} W_{p}\left(S^{c}\left(3,2m\right)\right) & = & \frac{\sum_{v\in V(S^{c}\left(3,2m\right))}\left|N_{S^{c}\left(3,2m\right)}^{3}\left(v\right)\right|}{2}\\ & = & \frac{(\sum_{i=1}^{2m}\left|N_{S^{c}\left(3,2m\right)}^{3}\left(a_{i}\right)\right|)\times 3}{2}\\ & = & 21m-27. \end{array}$$

This formula can be easily verified for *W*_*p*_(*S*^*c*^(3, 4)) = 15 (*m* = 2) and *W*_*p*_(*S*^*c*^(3, 6)) = 36 (*m* = 3).

**Case 2** If *n* = 4 and *m* ≥ 2; When *m* ≥ 4, we have

$$\begin{array}{c} |N_{S^{c}\left(4,2m\right)}^{3}\left(a_{i}\right)|=5\;\;\text{for}\;i=1,2,2m-1,2m; \end{array}$$

$$\begin{array}{c} |N_{S^{c}\left(4,2m\right)}^{3}\left(a_{3}\right)\left|=\right|N_{S^{c}\left(4,2m\right)}^{3}\left(a_{2m-2}\right)|=8; \end{array}$$

$$\begin{array}{c} |N_{S^{c}\left(4,2m\right)}^{3}\left(a_{i}\right)|=10\;\;\text{for}\;4\leq i\leq 2m-3. \end{array}$$

Thus

$$\begin{array}{ccc} W_{p}\left(S^{c}\left(4,2m\right)\right) & = & \frac{\sum_{v\in V(S^{c}\left(4,2m\right))}\left|N_{S^{c}\left(4,2m\right)}^{3}\left(v\right)\right|}{2}\\ & = & \frac{(\sum_{i=1}^{2m}\left|N_{S^{c}\left(4,2m\right)}^{3}\left(a_{i}\right)\right|)\times 4}{2}\\ & = & 40m-48. \end{array}$$

Again this can be verified for *m* = 2 or 3.

**Case 3** Similarly, we have

$$\begin{array}{c} W_{p}\left(S^{c}\left(n,2m\right)\right)=60m-65\;\;\text{for}\;n=5\;\;\text{and}\;m\geq 2; \end{array}$$

$$\begin{array}{c} W_{p}\left(S^{c}\left(n,2m\right)\right)=84m-78\;\;\text{for}\;n=6\;\;\text{and}\;m\geq 2. \end{array}$$

**Case 4** If *n* ≥ 7 and *m* ≥ 2;

- When *m* = 2 and *n* ≥ 7, we have
  $$\begin{array}{c} |N_{S^{c}\left(n,4\right)}^{3}\left(a_{1}\right)\left|=\right|N_{S^{c}\left(n,4\right)}^{3}\left(a_{4}\right)\left|=9;\;\;\;\right|N_{S^{c}\left(n,4\right)}^{3}\left(a_{2}\right)\left|=\right|N_{S^{c}\left(n,4\right)}^{3}\left(a_{3}\right)|=8. \end{array}$$
  Hence
  $$\begin{array}{ccc} W_{p}\left(S^{c}\left(n,4\right)\right) & = & \frac{\sum_{v\in V(S^{c}\left(n,4\right))}\left|N_{S^{c}\left(n,4\right)}^{3}\left(v\right)\right|}{2}\\ & = & \frac{(\sum_{i=1}^{4}\left|N_{S^{c}\left(n,4\right)}^{3}\left(a_{i}\right)\right|)\times n}{2}\\ & = & 17n. \end{array}$$
- When *m* = 3, we have
  $$\begin{array}{c} |N_{S^{c}\left(n,6\right)}^{3}\left(a_{1}\right)\left|=\right|N_{S^{c}\left(n,6\right)}^{3}\left(a_{6}\right)|=9; \end{array}$$
  $$\begin{array}{c} |N_{S^{c}\left(n,6\right)}^{3}\left(a_{2}\right)\left|=\right|N_{S^{c}\left(n,6\right)}^{3}\left(a_{5}\right)|=10; \end{array}$$
  $$\begin{array}{c} |N_{S^{c}\left(n,6\right)}^{3}\left(a_{3}\right)\left|=\right|N_{S^{c}\left(n,6\right)}^{3}\left(a_{4}\right)|=13. \end{array}$$
  and hence *W*_*p*_(*S*^*c*^(*n*, 6)) = 32*n*.
- When *m* ≥ 4, we have
  $$\begin{array}{c} |N_{S^{c}\left(n,2m\right)}^{3}\left(a_{1}\right)\left|=\right|N_{S^{c}\left(n,2m\right)}^{3}\left(a_{2m}\right)|=9; \end{array}$$
  $$\begin{array}{c} |N_{S^{c}\left(n,2m\right)}^{3}\left(a_{2}\right)\left|=\right|N_{S^{c}\left(n,2m\right)}^{3}\left(a_{2m-1}\right)|=10; \end{array}$$
  $$\begin{array}{c} |N_{S^{c}\left(n,2m\right)}^{3}\left(a_{3}\right)\left|=\right|N_{S^{c}\left(n,2m\right)}^{3}\left(a_{2m-2}\right)|=13; \end{array}$$
  $$\begin{array}{c} |N_{S^{c}\left(n,2m\right)}^{3}\left(a_{i}\right)|=15\;\;\text{for}\;4\leq i\leq 2m-3. \end{array}$$
  Hence
  $$\begin{array}{ccc} W_{p}\left(S^{c}\left(n,2m\right)\right) & = & \frac{\sum_{v\in V(S^{c}\left(n,2m\right))}\left|N_{S^{c}\left(n,2m\right)}^{3}\left(v\right)\right|}{2}\\ & = & \frac{(\sum_{i=1}^{2m}\left|N_{S^{c}\left(n,2m\right)}^{3}\left(a_{i}\right)\right|)\times n}{2}\\ & = & 15nm-13n. \end{array}$$

Note that the formula *W*_*p*_(*S*^*c*^(*n*, 2*m*)) = 15*nm* − 13*n* also hold for both *m* = 2 and *m* = 3.

**(iii)** Lastly, we calculate the Wiener polarity index of *S*^*t*^(*n*, 2*m*), the 3^3^ ⋅ 4^2^ lattice with toroidal boundary condition. As in *H*^*t*^(*n*, *m*) and *T*^*t*^(*n*, *m*), all vertices of *S*^*t*^(*n*, 2*m*) have the same number of 3rd neighbors. Also note that |*V*(*S*^*t*^(*n*, 2*m*))| = 2*nm*.

**Case 1** If *n* = 3 and *m* ≥ 2, it is easy to see that *W*_*p*_(*S*^*t*^(3, 4)) = 0 and *W*_*p*_(*S*^*t*^(3, 6)) = 45. When *m* ≥ 4, we have $|N_{S^{t}\left(3,2m\right)}^{3}\left(v\right)|=7$ for any *v* ∈ *V*(*S*^*t*^(3, 2*m*)). Hence

$$\begin{array}{ccc} W_{p}\left(S^{t}\left(3,2m\right)\right) & = & \frac{\sum_{v\in V(S^{t}\left(3,2m\right))}\left|N_{v}^{3}\left(v\right)\right|}{2}\\ & = & \frac{7\times |V(S^{t}\left(3,2m\right))|}{2}\\ & = & 21m. \end{array}$$

**Case 2** If *n* = 4 and *m* ≥ 2;

- When *m* = 2, we have *W*_*p*_(*S*^*t*^(4, 4)) = 16.
- When *m* = 3, we have *W*_*p*_(*S*^*t*^(4, 6)) = 108.
- When *m* ≥ 4, we have |*V*(*S*^*t*^(4, 2*m*))| = 8*m* and $|N_{S^{t}\left(4,2m\right)}^{3}\left(v\right)|=10$ for any *v* ∈ *V*(*S*^*t*^(4, 2*m*)). Hence *W*_*p*_(*S*^*t*^(4, 2*m*)) = 40*m* by Lemma 4.

**Case 3** If *n* = 5 and *m* ≥ 2, similarly we have

$$\begin{array}{c} W_{p}\left(S^{t}\left(5,4\right)\right)=50;\;\;\;W_{p}\left(S^{t}\left(5,6\right)\right)=165; \end{array}$$

and

$$\begin{array}{c} W_{p}\left(S^{t}\left(5,2m\right)\right)=60m\;\;\;\text{for}\;m\geq 4; \end{array}$$

**Case 4** If *n* = 6 and *m* ≥ 2, similarly we have

$$\begin{array}{c} W_{p}\left(S^{t}\left(6,4\right)\right)=84;\;\;\;W_{p}\left(S^{t}\left(6,6\right)\right)=234; \end{array}$$

and

$$\begin{array}{c} W_{p}\left(S^{t}\left(6,2m\right)\right)=84m\;\;\;\text{for}\;m\geq 4. \end{array}$$

**Case 5** If *n* ≥ 7 and *m* ≥ 2:

- When *m* = 2, we have $|N_{S^{t}\left(n,4\right)}^{3}\left(v\right)|=8$ for any *v* ∈ *V*(*S*^*t*^(*n*, 4)) and *W*_*p*_(*S*^*t*^(*n*, 4)) = 16*n*.
- When *m* = 3, we have $|N_{S^{t}\left(n,6\right)}^{3}\left(v\right)|=14$ for any *v* ∈ *V*(*S*^*t*^(*n*, 6)) and *W*_*p*_(*S*^*t*^(*n*, 6)) = 42*n*.
- When *m* ≥ 4, we have $|N_{S^{t}\left(n,2m\right)}^{3}\left(v\right)|=15$ for any *v* ∈ *V*(*S*^*t*^(*n*, 2*m*)) and hence
  $$\begin{array}{ccc} W_{p}\left(S^{t}\left(n,2m\right)\right) & = & \frac{\sum_{v\in V(S^{t}\left(n,2m\right))}\left|N_{S^{t}\left(n,2m\right)}^{3}\left(v\right)\right|}{2}\\ & = & \frac{15\times |V(S^{t}\left(n,2m\right))|}{2}\\ & = & 15nm. \end{array}$$

## Concluding remarks

Evaluation of topological indices of network structures is an important problem in the study of network robustness [71–73]. In particular, the computation of distance-based graph indices of various lattices has attracted the attention of researchers from many different backgrounds. By using a fundamental general formula of the Wiener polarity index of graphs, we determined the explicit formulas for the Wiener polarity index of the square lattices, the hexagonal lattices, the triangular lattices, and the 3^3^ ⋅ 4^2^ lattices with free, cylindrical and toroidal boundary conditions. The results of Theorems 2, 3, and 4 are plotted in Figs 4–6 respectively.

There exist other interesting graph structures of practical interests, such as the polyomino chains and the triangular Kagomé lattices. It would be worthwhile to explore their structure through computation of similar graph indices [74–77]. Given our findings, it may also be interesting to study the asymptotic behavior of a given topological index of these lattices structures. Furthermore, it could be a challenge to develop theoretical bounds on such indices, when certain restrictions (such as the sizes and number of “holes” in a hexagonal system) accommodated by the graph structure.
